# Functionally Improved Mesenchymal Stem Cells to Better Treat Myocardial Infarction

**DOI:** 10.1155/2018/7045245

**Published:** 2018-11-25

**Authors:** Zhi Chen, Long Chen, Chunyu Zeng, Wei Eric Wang

**Affiliations:** ^1^Department of Cardiology, Daping Hospital, Third Military Medical University, 10 Changjiang Branch Road, Chongqing 400042, China; ^2^College of Medicine, Soochow University, Suzhou 215123, China

## Abstract

Myocardial infarction (MI) is one of the leading causes of death worldwide. Mesenchymal stem cell (MSC) transplantation is considered a promising approach and has made significant progress in preclinical studies and clinical trials for treating MI. However, hurdles including poor survival, retention, homing, and differentiation capacity largely limit the therapeutic effect of transplanted MSCs. Many strategies such as preconditioning, genetic modification, cotransplantation with bioactive factors, and tissue engineering were developed to improve the survival and function of MSCs. On the other hand, optimizing the hostile transplantation microenvironment of the host myocardium is also of importance. Here, we review the modifications of MSCs as well as the host myocardium to improve the efficacy of MSC-based therapy against MI.

## 1. Introduction

Myocardial infarction (MI) leads to a massive loss of functional cardiomyocytes, which is a major cause of human death worldwide [1–3]. Though pharmacotherapy, thrombolysis, coronary stent implantation, and coronary artery bypass grafting have been clinically used to treat MI and improve patients' survival, these methods cannot fundamentally repair the damaged heart and restore heart function. Stem cell transplantation is considered as a promising way to treat MI, which has made significant progress in preclinical and clinical studies recently [4]. Stem cell candidates mainly include two categories: (1) pluripotent stem cells (embryonic stem cell and induced pluripotent stem cells) and their derivatives and (2) adult stem cells, including hematopoietic stem cells and mesenchymal stem cells (MSCs) [5]. MSCs are mesoderm-derived multipotent stromal cells that reside in embryonic and adult tissues, having the capacity for self-renewal, immune privilege, immunomodulation, and low tumorigenicity [6]. To date, MSCs have become the mostly practiced cell type in clinical trials for treating MI [7], due to the safety, multidifferentiation potential, nutritional activity, immunomodulatory properties, and abundant donor sources [6, 8]. MSCs have low immunogenicity due to the low expression of MHC II as well as the lack of expression of MHC I, which lead to immune tolerance allowing allogeneic transplantation [8].

However, the therapeutic effect of MSC transplantation is unsatisfactory. The increase in left ventricular systolic function (LVSF) of MI patients is only 3–10% with MSC transplantation [9]. Implanted cells do not survive for a long time. In fact, only about 3% of MSCs appeared in the marginal area of the infarct myocardium within 24 hours after systemic administration, and less than 1% of MSCs could survive for more than a week [5]. Recent studies have concluded that MSCs are very difficult to differentiate towards cardiomyocytes, and the benefits of MSC therapy mainly depend on its paracrine mechanism [10]. The key steps of the cell therapy procedures, such as donor selection, *in vitro* amplification, survival in a hostile transplantation microenvironment, migration, differentiation, and paracrine function, need to be optimized. Here, we review the strategies of MSC modifications for optimizing the therapeutic potential of MSCs against MI.

## 2. Therapeutic Effect of MSCs against MI Injury

MSCs have the potential of self-renewal, proliferation, and multidifferentiation in an appropriate microenvironment [11]. MSCs exert a therapeutic effect on MI through direct differentiation into vessel cells (cardiomyocyte differentiation events are rare) and paracrine mechanism (which has been proved predominant) [10]. Transplanted MSC-derived endothelial cells and vascular smooth muscle cells can contribute to the new vessel formation [12–14]. MSC paracrine factors include protein cytokines such as vascular endothelial growth factor (VEGF), hepatocyte growth factor (HGF), insulin-like growth factor (IGF), miRNAs [15–17], and exosomes [18]. These factors can induce immunomodulation and anti-inflammatory effects, evidenced by inhibition of the activity of inflammatory mediators and regulation of the function of immune cells [19]. The factors can induce an antifibrosis effect by inhibiting the proliferation of fibroblasts, reducing the deposition of collagen and producing matrix metalloproteinases [20]. In addition, factors such as stromal cell-derived factor-1 (SDF-1), VEGF, and basic fibroblast growth factor (bFGF) have a strong proangiogenic effect, due not only to promotion of endothelial cell proliferation and migration but also to prevention of endothelial cells from apoptosis [8, 21].

The MSC-based treatments for MI have successfully entered phase I and phase II clinical trials. A meta-analysis comprising 34 randomized controlled trials (RCTs) with a total number of 2307 patients indicates that MI patients who received MSC transplantation showed a significantly improved cardiac function, a significant increase in the left ventricular ejection fraction (LVEF) (+3.32%), and a decrease in LV end-diastolic indexes (−4.48) and LV end-systolic indexes (−6.73) [22]. Another meta-analysis covering 28 RCTs with a total of 1938 STEMI patients shows that MSC treatment resulted in an improvement in long-term (12 months) LVEF of 3.15% [23]. A recent study also showed benefits of MSC transplantation on mechanical and clinical outcomes. The LVEF of MI patients with MSC treatment increased by 3.84%, and the effect was maintained for up to 24 months. Scar mass was reduced by −1.13, and the wall motion score index was reduced by −0.05 at 6 months after MSC treatment [24]. Clinical trials of MSC transplantation for treating MI are listed in Table 1. Though previous clinical trials have made some advances, optimizing the process of MSC transplantation is needed in preparing for the clinical phase III trials.

## 3. Strategies for Optimizing MSC-Based Therapy

MSCs can be obtained from various tissues such as bone marrow, fat, peripheral blood, lungs, muscle, placenta, umbilical cord blood, and dental pulp [40]. Bone marrow MSCs (BM-MSCs) are the most frequently investigated and tested in clinical trials. It is reported that MSCs from younger donors are more effective than those from older donors, indicating an age-dependent effect of MSC functions. The expressions of inhibitory kappa B kinase, interleukin-1a, and inducible nitric oxide synthase in the elderly donor's MSCs were significantly decreased [15]. Previous studies showed that the expression of the pigment epithelium-derived factor (PEDF) was significantly increased in MSCs of aged mice compared with young mice. Knockout PEDF in aged MSCs can improve the therapeutic effect of MSCs [41]. These data suggest that using young MSCs for treating MI might be a more advisable option.

For cell number of MSC transplantation, ~10^5^–10^8^ MSCs were reported in diverse studies [42], but usually >1 × 10^7^ cells were required in clinical trials given the low retention rate [43, 44]. Cell expansion *in vitro* is needed for about 1–3 months before implantation to obtain enough cell numbers [5]. However, cell aging and the loss of chemokine markers during amplification could reduce the cell survival and functions of MSCs in the transplantation microenvironment. Methods such as environmental preconditioning, cytokine or drug coculture, and gene modification may overcome these problems.

### 3.1. Preconditioning MSCs in Culture before Transplantation

#### 3.1.1. Hypoxia Preconditioning

The peripheral area of MI is a typical site for preclinical MSC treatment. The oxygen partial pressure in the peripheral area generally does not exceed 1%, and hypoxia is a major cause of dysfunction and death of transplanted MSCs [45]. Hypoxia preconditioning *in vitro* (2–5% oxygen) can maintain homogeneity and differentiation potential, delay cell senescence process, and increase chemokine receptor expression of MSCs [46]. Hypoxia preconditioning is also proved to increase the paracrine activity of nonhuman primate MSCs [47]. Thus, MSCs with hypoxia preconditioning is more therapeutically effective against massive myocardium injury and does not increase the incidence of arrhythmia complications [48].

#### 3.1.2. Hyperoxia or Hydrogen Peroxide Preconditioning

Hyperoxia pretreatment can also improve MSC efficacy by reducing the number of apoptotic cells. BM-MSCs were implanted into hypoxic tissues after hyperoxia (100% oxygen), and the apoptotic cells were significantly reduced (apoptotic score index determined by TUNEL assays reduced from 86.6% to 11.6%) [49]. In addition, sublethal hydrogen peroxide preconditioning attenuated oxidative stress-induced cell apoptosis. Pretreatment with 200 *μ*mol/L H_2_O_2_ for 2 hours decreased MSC apoptosis. Compared with control MSCs, MSCs with H_2_O_2_ pretreatment better improved cardiac function and reduced myocardial fibrosis [50].

#### 3.1.3. Thermal Preconditioning

MSCs were incubated with water bath at 42°C for 2 hours before transplantation can effectively reduce the oxide-induced apoptosis of MSCs and enhance cell survival. The mechanism may be related to the expression of heat shock proteins, which act as a molecular chaperone and indirectly promote cell survival by inhibiting the apoptosis pathway and resist oxidation stress [51].

#### 3.1.4. Nutritional Deprivation Preconditioning

The transplantation microenvironment is poor in nutritional supply. Reducing energy requirements to allow MSCs to enter a relatively quiescent state helps them adapt to the upcoming low-energy environment. Serum deprivation for 48 hours could induce MSCs into a quiescent state and improve MSC survival rates. Compared with control, serum deprivation increased the survival rates by 3–4-fold after the third day and on the seventh day after transplantation [52].

#### 3.1.5. Transient Adaptation Preconditioning

Although MSC itself is with low immunogenicity, the presence of immunogenic contamination in xenogeneic serum may result in acute rejection with the host immune system after MSC transplantation [53]. A two-stage culture strategy was developed to overcome this problem. In the first phase, the MSCs were isolated and expanded in the human platelet lysate or mixed allogeneic serum medium. Then, in the second stage, the expanded MSCs were cultured in the autologous serum medium. This transient adaptation in autologous serum may contribute to the expression of chemokine receptors and tissue-specific differentiation of amplified MSCs *in vitro*, which provides an efficient method for the immunological rejection [46].

### 3.2. Genetic Modification and Cytokine/Drug Treatment on MSCs

To obtain enough cell numbers, MSC expansion in culture usually needs 1–3 months [5]. Not only is the process time-consuming and laborious but also it is difficult to maintain the multidifferentiation ability. Viral vectors or nonviral methods were used to genetically modify MSCs before transplantation. Overexpression of antiapoptotic transcription factor Akt could significantly increase MSC viability [54]. MSCs transfected with both OCT4 and SOX2 showed a strong proliferative activity [55]. Overexpressing manganese superoxide dismutase can endow cells with anoxic tolerance before transplantation then effectively increase the survival rate [56]. Studies that enhance cell engraftment via genetic modification are listed in Table 2. Pretreating MSCs with cytokines/drugs prior to transplantation can promote cell proliferation. A combination of hypoxia (5% O_2_) and 10 ng/mL basic fibroblast growth factor generated a significant synergistic effect. It produced highly reproducible MSCs, allowing MSCs to maintain multidifferentiation ability after the 11th generation. Besides, the cell production is 2.8 times faster than the traditional method [57]. Chemical drugs are also used for MSC pretreatment. Proline hydroxylase inhibitor DMOG-pretreated MSCs significantly reduced cell mortality after transplantation, which is associated with elevated expressions of hypoxia-inducible factor-1*α* (HIF-1*α*), VEGF, GLUT-1, and phospho-Akt were significantly increased [58]. Mitochondrial electron transport inhibitors, such as antimycin, have been used to block the activation of mitochondrial death pathways [53]. Omentin-1 promotes MSC proliferation, inhibits apoptosis, increases the secretion of angiogenic cytokines, and enhances angiogenesis via the PI3K/Akt signaling pathway [59]. Studies that enhance cell engraftment via drug/cytokine pretreatment are listed in Table 3.

### 3.3. Cotransplantation MSCs with Bioactive Factors

Multiple studies have shown that cotherapy with drugs/specific cells/cytokines/specific biomaterials can prolong the survival time of MSCs and thus improve their therapeutic efficacy [117]. MSC transplantation combined with heparin significantly reduced the retention of MSCs in the lungs. Cotransplantation of MSCs and HGF improved cardiac function and reduced infarct size of post-MI heart [118]. Encapsulating cells in an injectable biomaterial could play an antioxidant role [119]. In a rat MI model, the survival rate of MSCs was increased by about 30% after coinjection with fibrin glue [120]. In a swine MI model, cotransplantation of MSCs and cardiac stem cells was reported to be superior than transplantation of each single type of stem cells [121]. Combined therapy of MSCs and rosuvastatin reduced fibrosis, decreased cardiomyocyte apoptosis, and preserved heart function [122]. Nutrient-rich plasma containing high levels of growth factors and secreted proteins has been identified as a biological material which can promote MSC function and promote wound healing. Thus, cotransplantation of MSCs with plasma is beneficial for MSCs adapting to nutritional deficiency in the infarct myocardium, which has been applied for clinical trials [123]. When we injected the MSCs through intravenous administration, it is easy to induce the block of vessels. Then, the use of vasodilator drugs significantly avoids the issues and contributes to the migration and homing of MSCs [53].

### 3.4. Biomaterials, Scaffolds, and Tissue Engineering to Improve MSC Functions

Long-term retention in the injection site is a necessary condition for the continued effectiveness of MSCs in the MI treatment. MSCs have multiple administration routes applied to clinical or preclinical studies. Injection routes including intravenous injection, intracoronary injection, intramyocardial injection (including transendocardial and transepicardial) were applied for MSC transplantation [124, 125]. Systematic intravenous injection is obviously simple and easy for dose control, but it causes massive cell redistribution into other organs such as the liver and lung. To date, intracoronary injection is the most studied technique during the time of percutaneous coronary intervention after MI, which is convenient and proved safe. Stem cells delivered through this method have been proved to improve cardiac function and reduce infarct size. Furthermore, specific studies comparing the effectiveness of different cell delivery routes showed that catheter-based transendocardial injection is superior to intracoronary injection, in terms of cell retention and cardiac function improvement [126]. Accumulating evidence supported that both transendocardial and surgical transepicardial injections are safe and effective in various preclinical and clinical studies [38]. Therefore, intramyocardial injection is considered to be the most efficient way for cell delivery [127]. However, even after intramyocardial delivery, the majority of transplanted cells are lost; thus, the above methods still could not guarantee the cell survival and long-term retention.

#### 3.4.1. Multicellular Spheres

Cell preparations based on multicellular spheres have proved to be a promising way to enhance the therapeutic potential of MSCs [128]. Compared with the traditional two-dimensional (2D) monolayer culture, three-dimensional (3D) cell tissue can enhance the intracellular effect. Compared with the same number of MSCs in the traditional 2D monolayer culture, the MSC sphere in fibrin gel increased the level of VEGF secretion by 100 times [129] and the level of the CXCR4 receptor by 2 times [130]. The MSC sphere also obviously increases the expressions of HIF-1, FGF2, HGF, and miRNAs related to pleiotropia [17, 131]. Therefore, 3D MSCs improve the anti-inflammatory and angiogenic properties of MSCs after transplantation. In both rodent and porcine MI models, 3D MSCs were shown to be differentiated into endothelial cells and myocardium-like cells after transplantation and improve cardiac function of post-MI hearts [132, 133].

#### 3.4.2. Cell Sheet and Hydrogels

Cell sheet technology has been confirmed to prolong the resident time of transplanted cells in the infarct myocardium [134]. The effect of three-layer MSC sheet administration for MI treatment is better than that of conventional intramyocardial injection [135]. The use of biomaterials, such as suspending MSCs in hydrogels or coated MSCs with hydrogel, may effectively reduce the mechanical forces during injection and protect cells from damage [136].

The process of survival and retention of MSCs can be affected by various factors, such as ischemia, hypoxia, and inflammatory cell attack. The application of tissue engineering can improve this undesirable state [137]. The physical properties and microstructure of hydrogels regulate the infiltration of inflammatory cytokines and T lymphocytes *in vivo*, thereby reducing the attack of inflammatory cells on MSCs [53]. Injecting MSCs in an in situ cross-linked alginate hydrogel can maintain its activity and keep its paracrine with no immunogenicity [138]. Encapsulating MSCs in an alginate hydrogel patch may also improve the retention of MSCs [139]. The collagen scaffolds (such as type I collagen scaffolds) can enhance the adhesion and proliferation of MSCs and exhibit better cytocompatibility [4].

In addition, the invention of an artificial simulated extracellular matrix based on tissue engineering has overcome many difficulties in the application of MSCs. Using hydrogels as scaffolds and adding high-affinity growth factors and chemokines may overcome the loss of chemokines via cell-scaffold interaction [4, 140]. MSCs suspended at 2% sodium alginate (a natural hydrogel) before transplanting was four times more effective [141].

#### 3.4.3. Nanomaterials

Nanobiomaterial-incorporated stem cell therapy for MI has aroused much attention in recent years. The cardiac patch [142], nanofibrous scaffolds [143], and self-assembling peptides [144] appear promising in repairing the damaged myocardium. Cardiac patches consist of native collagen or synthetic polymers with a nanofibrous structure poly(lactide-co-epsilon caprolactone (PLCL)). These patches function when they are placed on the epicardial surface of the infarcted myocardium. PLCL is a highly flexible polymer which can form nanofibrous scaffolds, which significantly improves the survival rate of implanted MSCs compared to MSCs by direct injection [145]. Bioinspired self-assembling peptide nanofibers can be used as a cell carrier. MSCs that dealt with functional self-assembling peptide nanofibers RAD/PRG or RAD/KLT showed improved efficacy to treat MI [144]. Another study constructed poly(lactide-co-glycolide)-monomethoxy-poly-(polyethylene glycol) nanoparticles to encapsulate melatonin on adipose-derived MSCs and improve the efficiency of their transplantation [146].

### 3.5. Modifying Transplantation Environment of the Host Myocardium

Modifying the target tissue prior to MSC transplantation to make the environment more conducive is a supplement approach to donor cell pretreatment. C1q/tumor necrosis factor-related protein-9 (CTRP9) is a novel prosurvival cardiokine with a significantly downregulated expression after MI, which is critical in maintaining a healthy microenvironment facilitating stem cell engraftment in infarcted myocardial tissue. Overexpression of CTRP9 in the host myocardium significantly enhanced stem cell therapeutic efficacy [147].

The process of transporting MSCs to damaged tissue is called homing, which is the result of the interaction of multiple chemokines and their receptors. CXC chemokine receptor 4 (CXCR4) and SDF-1 play a key role in the homing. MSCs are naturally capable of migrating to the injured area in the myocardium, but this feature is impaired because *in vitro* culture would induce the loss of the key homing receptor CXCR4 and other cellular signals. Releasing the adenoviruses carrying SDF-1*α* to increase the local concentration of SDF-1*α* in the injured myocardium could increase the homing of MSCs [90]. Combination of SDF-1 secretes from the infarct myocardium, and CXCR4 in MSCs can induce the migration of MSCs to the injured site [4]. Meanwhile, transfection of MSCs with CXCR4 overexpression vector increased the number of migrating MSCs by 3-fold [4].

### 3.6. Novel Approaches to Stimulate MSC Homing

Another intriguing method to increase the homing efficiency of MSCs is cell surface engineering, which is the temporary modification of the cell surface. These temporary changes help to improve the homing of MSCs without affecting viability, proliferation, adhesion, or differentiation of the transplanted cells [148]. In addition, the phage display approaches were used to screen MI-specific peptide sequences. In MI mouse models, four peptide sequences (CRPPR, CRKDKC, KSTRKS, and CARSKNKDC4) were identified. The number of homing MSCs was significantly increased by injecting MSCs coated with MI-specific homing peptide in treating MI, indicating that the use of homing peptide-coated MSCs is a promising method for the treatment of MI [149].

Except for molecular modification of MSCs, it has been found that radiation, ultrasound, electric field, or magnetic field can also promote homing. Within 4 hours of MI, treating the bone marrow with 804 nm wavelength and 1 J/cm^2^ energy density can increase the survival, proliferation, and homing of MSCs [150]. The magnetic targeting technique (MTT) is based on the premagnetization of MSCs and then MSCs move *in vivo* with the aid of a magnetic field [151]. MTT allows a wider range of transplanted cells to reach the target tissue, providing a more efficient and sustained medium release without increasing the number of MSCs [152].

## 4. Conclusion and Future Perspectives

**Figure 1:**
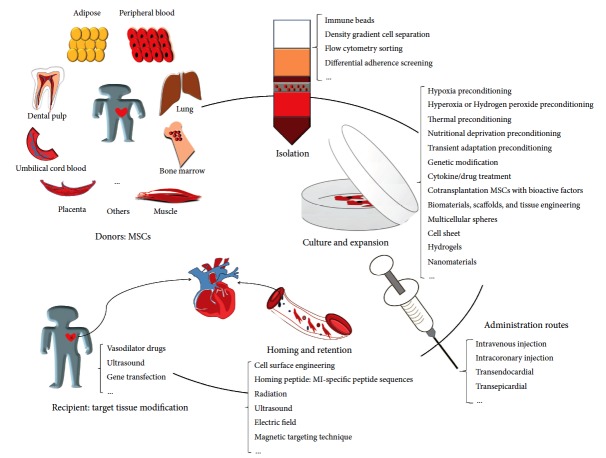
The procedures of MSC-based therapy, including donor selection, cell expansion, dosage, injection routes, homing, and target tissue modification. MSCs: mesenchymal stem cells.

Many strategies were developed to modify the MSCs as well as the transplantation microenvironment, which improve the survival, retention, homing, multidifferentiation capacity, and paracrine factors, thereby enhancing the outcome of MSC-based therapy against MI (Figure 1). The combination of certain methods may exert synergistic effects to improve the efficacy of MSC transplantation. Clinical trials have shown that MSC transplantation is feasible and safe for MI, and it does not increase the risk of adverse events. Although some approaches such as supplement with rosuvastatin are clinically safe [122], whether other methods to improve the MSC functions are safe when applying to patients is currently uncertain. Further optimizing these methods to achieve clinical safety and effectiveness is of great significance for stem cell therapy.

## Figures and Tables

**Table 1:** Clinical trials of MSC transplantation for treating MI.

Clinical trials	Phase	Dose (^∗^10^6^)	Delivery route	Enrollment	Infarct scar	LVEF	Following up	Study	Reference
NCT00114452	Phase 1	0.5/1.6/5	IC	53	n.a.	↑ ^∗∗^	6 m	Hare et al. (2009)	[25]
NCT00677222	Phase 1	100	IC	30	n.a.	↑ ^∗^	4 m	Penn et al. (2012)	[26]
2011AA020109	Phase 1	3.08	IC	43	=	↑ ^∗^	12 m	Gao et al. (2013)	[27]
UO1 HL087318–04	Phase 1	150	IC	65	↓ ^∗^	↑ ^∗∗∗^	12 m	Traverse et al. (2014)	[28]
NCT01234181	Phase 1	100	IC	22	↓ ^∗^	↑ ^∗^	12 m	Hu et al. (2015)	[29]
NCT01087996	Phase 1/2	20	IM	30	↓^∗∗∗^	↑	13 m	Hare et al. (2012)	[30]
U54HL081028	Phase 1/2	20	IM	30	↓^∗^	↑ ^∗∗^	13 m	Suncion et al. (2014)	[31]
NCT02323477	Phase 1/2	20	IM	79	n.a.	n.a.	12 m	Can et al. (2015)	[32]
NCT00883727	Phase 1/2	180–220	IV	20	=	=	2 y	Chullikana et al. (2015)	[33]
NCT02504437	Phase 1/2	—	—	200	—	—	12 m	Pei (2015–2017)	ClinicalTrials.gov
NCT02503280	Phase 1/2	200	—	55	—	—	12 m	Joshua (2015–2032)	ClinicalTrials.gov
NCT02666391	Phase 1/2	—	—	64	—	—	18 m	Pei (2016–2017)	ClinicalTrials.gov
NCT01770613	Phase 2	—	—	—	—	—	12 m	Nabil (2013–2017)	ClinicalTrials.gov
NCT00684021	Phase 2	150	IC	101	n.a.	↑ ^∗∗∗^	6 m	Schutt et al. (2015)	[34]
NCT00984178	Phase 2	15	IC	120	↓ ^∗^	↑ ^∗∗^	12 m	San Roman et al. (2015)	[35]
NCT00765453	Phase 2	59.8	IC	100	n.a.	↑ ^∗∗∗^	12 m	Choudry et al. (2015)	[36]
NCT01291329	Phase 2	6	IC	116	↓ ^∗∗∗^	↑ ^∗∗∗^	18 m	Gao et al. (2015)	[37]
NCT03047772	Phase 2	—	—	124	—	—	12 m	Yang (2017–2018)	ClinicalTrials.gov
NCT00877903	Phase 2	—	IV	220	—	—	5 y	Donna (2009–2018)	ClinicalTrials.gov
NCT02013674	Phase 2	100	IM	30	↓^∗^	↑ ^∗^	12 m	Florea et al. (2013–2019)	[38]
NCT01392105	Phase 2/3	72	IC	80	n.a.	↑ ^∗^	6 m	Lee et al. (2014)	[39]
NCT03404063	Phase 2/3	30		115	—	—	6 m	Piotr (2017–2020)	ClinicalTrials.gov
NCT01394432	Phase 3	—	IM	50	—	—	12 m	Evgeny (2012–2016)	ClinicalTrials.gov
NCT01652209	Phase 3	—	—	135	—	—	13 m	Yang (2013–2020)	ClinicalTrials.gov
NCT02672267	Phase 3	—	IM	50	—	—	6 m	Saule (2014–2016)	ClinicalTrials.gov

MSCs: mesenchymal stem cells; MI: myocardial infarction; IM: intramyocardial; IC: intracoronary; IV: intravenous; LVEF: left ventricular ejection fraction; y: year; m: month; n.a.: not analyzed; =: no statistical significance. ^∗^*p* < 0.05, ^∗∗^*p* < 0.01, and ^∗∗∗^*p* < 0.001.

**Table 2:** Gene modification in MSC transplantation for treating MI.

Gene name	Disease	Model	Modification	Gene function	Reference
Hsp27	MI	Rat	Overexpression	Viability↑; apoptosis↓	[60]
MicroRNA-133	MI	Rat	Overexpression	Survival ↑	[61]
SDF-1*α*	MI	Rat	Overexpression	Homing↑	[62]
CAMKK1	MI	Rat	Overexpression	Angiogenesis↑; infarct size↓; ejection fraction↑	[63]
eNOS	MI	Rat	Overexpression	Infarct size↓; angiogenesis↑	[64]
Akt1	MI	Rat	Overexpression	Cardiac function↑	[45]
PKG1*α*	MI	Rat	Overexpression	Survival↑; angiogenesis↑	[65]
Caspase 8	MI	Rat	Silence	Cardiac fibrosis↓; survival↑	[66]
SIRT1	MI	Rat	Overexpression	Cardiac remodeling↓; angiogenesis↑	[67]
Netrin-1	MI	Rat	Overexpression	Survival↑; migration	[68]
FGF4-bFGF	MI	Rat	Overexpression	Survival↑; microvascular density↑; cardiac fibrosis↓	[69]
MicroRNA-377	MI	Rat	Knockdown	Angiogenesis↑	[70]
PKC*ɛ*	MI	Rat	Overexpression	Survival ↑; infarct size ↓apoptosis ↓	[71]
Trx1	MI	Rat	Overexpression	Angiogenesis↑	[72]
BCL2L1 (Bcl-xL)	MI	Rat/MSC culture	Overexpression	Apoptosis↓; angiogenesis↑	[73]
MDK	MI	Rat/MSC culture	Overexpression	Apoptosis↓; cardiac function↑	[74]
miR-23a	MI	Rat/MSC culture	Overexpression	Apoptosis↓; infarct size↓	[75]
miR Let-7b	MI	Rat/MSC culture	Overexpression	Cardiac function↓; infarct size↓; angiogenesis ↑	[76]
VEGF	MI	Rat/MSC culture	Overexpression	Survival ↑; angiogenesis ↑	[77]
HIF-1A	MI	Rat/MSC culture	Overexpression	Paracrine↑; angiogenesis↑; migration↑	[78]
KLK1 (tissue kallikrein)	MI	Rat/MSC culture	Overexpression	Apoptosis ↓; apoptosis↓	[79]
PHD2	MI	Mouse	Silence	Survival↑; apoptosis↓;scar size↓	[80]
ecSOD	MI	Mouse	Overexpression	Infarction size↓; apoptosis↓; survival.↑	[81]
MIR1-1 (miR-1)	MI	Mouse	Overexpression	Survival↑	[82]
HGF	MI	Mouse	Overexpression	Angiogenesis↑; apoptosis↓	[83]
ILK	MI	Porcine	Overexpression	Homing↑; LVEF↑; myocardial remodeling↓	[84]
IGF-1	MI	Porcine	Overexpression	Angiogenesis ↑	[85]
GLP-1	MI	Porcine	Overexpression	Angiogenesis ↑	[86]
VEGF (165)	MI	Ovine	Overexpression	Infarct size↓; left ventricular function↑	[87]
hRAMP1	MI	Rabbit	Overexpression	Infarct size↓	[88]
SOD2	—	MSC culture	Overexpression	Apoptosis↓	[56]
miR-210	—	MSC culture	Overexpression	Apoptosis↓; survival↑	[88]
CXCL12	—	MSC culture	Overexpression	Apoptosis↓; proliferation↑	[89]

MDK: midkine; Trx1: thioredoxin-1; PKC*ɛ*: protein kinase C *ɛ*; IGF-1: insulin-like growth factor-1; Hsp27: exogenous heat shock protein 27; SOD2: manganese superoxide dismutase; OH-1: heme oxygenase; CXCR4: CXC chemokine receptor 4; CAMKK1: calcium/calmodulin-dependent protein kinase kinase-1;eNOS:endothelial nitric oxide synthases; ILK: integrin-linked kinase; Nrf2: nuclear factor- (erythroid-derived 2-) like 2; PHD2: prolyl hydroxylase domain protein 2; GLP-1: glucagon-like peptide-1; SIRT1: silent mating type information regulation 2 homolog 1; FGF4: fibroblast growth factor 4; bFGF: basic fibroblast growth factor; ecSOD: extracellular superoxide dismutase; RAMP1: receptor activity-modifying protein 1; PKG1*α*: protein kinase type 1*α*.

**Table 3:** Drug/cytokine pretreatment in MSC transplantation for treating MI.

Drug/cytokine	Disease	Model	Dose/method	Function	Reference
Pioglitazone	MI	Rat	3 mg/kg/day/2 weeks	Cardiac function ↑	[90]
Atorvastatin	MI	Rat	1 mM/24 h	Neovascularization ↑	[91]
Sevoflurane	MI	Rat	3%/30 min	Activation of CSCs	[16]
Tadalafil	MI	Rat	1 *μ*mol/L/2 h	Survival ↑; homing ↑	[92]
AER-ME	MI	Rat	200 mg/kg/day/30 days	Viability ↑; differentiation ↑	[93]
SRT1720	MI	Rat	0.5 *μ*M/24 h	Survival ↑	[94]
Angiotensin II	MI	Rat	100 nM/24 h	Infarct size ↓	[95]
Salvianolic acid B	MI	Rat	10 *μ*M/30 min	Infarct size ↓	[96]
DNP	MI	Rat	0.25 mM/20 min	Infarct size ↓; angiogenesis ↑	[97]
Edaravone	MI	Rat	500 *μ*M	Apoptosis ↓; migration ↑	[98]
Trimetazidine	MI	Rat	2.08 mg/kg/day	Apoptosis ↓;infarct size ↓	[99]
IGF-1	MI	Rat	10 ng/mL/48 h	Survival ↑; apoptosis ↓	[100]
IL-1*β*, TNF-*α*	MI	Rat	10 ng/mL/24 h	Infarct size ↓	[101]
(EGb) 761	MI	Rat	100 mg/kg/day	Antioxidant ↑; differentiation ↑	[102]
T*β*4	MI	Rat	1 *μ*g/mL/48 h	Proliferation ↑; retention ↑; survival ↑	[103]
Tanshinone IIA	MI	Rat	0.2 *μ*g/mL/72 h	Migration ↑	[104]
Astragaloside IV	MI	Rat	0.4 *μ*g/mL/72 h	Migration ↑	[104]
Melatonin	MI	Mouse	5 mM/24 h	Infarct size ↓	[105]
Apicidin	MI	Mouse	3 *μ*M/24 h	Cardiac markers ↑	[106]
H_2_O_2_	MI	Mouse	200 *μ*mol/L/2 h	Apoptosis ↓; angiogenesis ↑	[50]
PMSNs-siCCR2	MI	Mouse	25 *μ*g/g/cotransplantation	Survival ↑; angiogenesis ↑	[107]
Aliskiren	MI	Mouse	15 mg/kg/day	Survival ↑; systolic function ↑	[108]
Atorvastatin	MI	Porcine	0.25 mg/kg/day	Infarct size ↓	[109]
TG-0054	MI	Porcine	2.85 mg/kg/day	LV contractility ↑	[110]
GLP-1	MI	Porcine	100 nM/48 h	Apoptosis ↓; infarct size ↓	[111]
G-CSF	MI	Rabbit	20 u/kg/day	Apoptosis ↓	[112]
Atorvastatin	MI	Rabbit	1.5 mg/kg/day	Myocardial remodeling ↓	[113]
Nicorandil	—	MSC culture	100 *μ*M/1.5 h	Apoptosis ↓	[114]
Geraniin	—	MSC culture	20 *μ*M/24 h	Efficacy ↑	[115]
Exendin-4	—	MSC culture	0–20 nm/L/12 h	Proliferation ↑	[116]

DNP: 2,4-dinitrophenol; GLP-1 :glucagon-like peptide-1; DMOG: dimethyloxalyl glycine; AER-ME: Ailanthus excelsa Roxb. methanolic extract; PMSNs: siRNA-loaded photoluminescent mesoporous silicon nanoparticles. TG-0054: a novel CXCR4 antagonist; EGb 761: Ginkgo biloba extract; G-CSF: granulocyte colony-stimulating factor; T*β*4: thymosin *β*4.

## Abbreviations

MI: Myocardial infarction
MSCs: Mesenchymal stem cells
BM-MSCs: Bone marrow mesenchymal stem cells
LVEF: Left ventricular ejection fraction
LVSF: Left ventricular systolic function
VEGF: Vascular endothelial growth factor
HGF: Hepatocyte growth factor
IGF-1: Insulin-like growth factor-1
SDF-1: Stromal cell-derived factor-1
PEDF: Pigment epithelium-derived factor
bFGF: Basic fibroblast growth factor
RCTs: Randomized controlled trials
HIF-1*α*: Hypoxia inducible factor-1*α*.

## References

[1] GBD 2013 Mortality and Causes of Death Collaborators (2015). Global, regional, and national age-sex specific all-cause and cause-specific mortality for 240 causes of death, 1990-2013: a systematic analysis for the Global Burden of Disease Study 2013. *Lancet*.

[2] Samsonraj R. M., Raghunath M., Nurcombe V., Hui J. H., van Wijnen A. J., Cool S. M. (2017). Concise review: multifaceted characterization of human mesenchymal stem cells for use in regenerative medicine. *Stem Cells Translational Medicine*.

[3] Go A. S., Mozaffarian D., Roger V. L. (2014). Heart Disease and Stroke Statistics—2014 Update: a report from the American Heart Association. *Circulation*.

[4] Ji S. T., Kim H., Yun J., Chung J. S., Kwon S. M. (2017). Promising therapeutic strategies for mesenchymal stem cell-based cardiovascular regeneration: from cell priming to tissue engineering. *Stem Cells International*.

[5] Vizoso F., Eiro N., Cid S., Schneider J., Perez-Fernandez R. (2017). Mesenchymal stem cell secretome: toward cell-free therapeutic strategies in regenerative medicine. *International Journal of Molecular Sciences*.

[6] Sanina C., Hare J. M. (2015). Mesenchymal stem cells as a biological drug for heart disease: where are we with cardiac cell-based therapy?. *Circulation Research*.

[7] Lee S., Choi E., Cha M. J., Hwang K. C. (2015). Cell adhesion and long-term survival of transplanted mesenchymal stem cells: a prerequisite for cell therapy. *Oxidative Medicine and Cellular Longevity*.

[8] Miao C., Lei M., Hu W., Han S., Wang Q. (2017). A brief review: the therapeutic potential of bone marrow mesenchymal stem cells in myocardial infarction. *Stem Cell Research & Therapy*.

[9] Liu B., Duan C. Y., Luo C. F. (2014). Effectiveness and safety of selected bone marrow stem cells on left ventricular function in patients with acute myocardial infarction: a meta-analysis of randomized controlled trials. *International Journal of Cardiology*.

[10] Maguire G. (2014). Stem cell therapy without the cells. *Communicative & Integrative Biology*.

[11] Nagamura-Inoue T., He H. (2014). Umbilical cord-derived mesenchymal stem cells: their advantages and potential clinical utility. *World Journal of Stem Cells*.

[12] Loffredo F. S., Steinhauser M. L., Gannon J., Lee R. T. (2015). Bone marrow-derived cell therapy stimulates endogenous cardiomyocyte progenitors and promotes cardiac repair. *Cell Stem Cell*.

[13] White I. A., Sanina C., Balkan W., Hare J. M. (2016). Mesenchymal stem cells in cardiology. *Methods in Molecular Biology*.

[14] Wang W. E., Yang D., Li L. (2013). Prolyl hydroxylase domain protein 2 silencing enhances the survival and paracrine function of transplanted adipose-derived stem cells in infarcted myocardium. *Circulation Research*.

[15] Pandey A. C., Lancaster J. J., Harris D. T., Goldman S., Juneman E. (2017). Cellular therapeutics for heart failure: focus on mesenchymal stem cells. *Stem Cells International*.

[16] Wen T., Wang L., Sun X. J., Zhao X., Zhang G. W., Li-Ling J. (2017). Sevoflurane preconditioning promotes activation of resident CSCs by transplanted BMSCs via miR-210 in a rat model for myocardial infarction. *Oncotarget*.

[17] Guo L., Zhou Y., Wang S., Wu Y. (2014). Epigenetic changes of mesenchymal stem cells in three-dimensional (3D) spheroids. *Journal of Cellular and Molecular Medicine*.

[18] Suzuki E., Fujita D., Takahashi M., Oba S., Nishimatsu H. (2017). Therapeutic effects of mesenchymal stem cell-derived exosomes in cardiovascular disease. *Advances in Experimental Medicine and Biology*.

[19] di Nicola M., Carlo-Stella C., Magni M. (2002). Human bone marrow stromal cells suppress T-lymphocyte proliferation induced by cellular or nonspecific mitogenic stimuli. *Blood*.

[20] Karantalis V., Hare J. M. (2015). Use of mesenchymal stem cells for therapy of cardiac disease. *Circulation Research*.

[21] Burlacu A., Grigorescu G., Rosca A. M., Preda M. B., Simionescu M. (2013). Factors secreted by mesenchymal stem cells and endothelial progenitor cells have complementary effects on angiogenesis in vitro. *Stem Cells and Development*.

[22] Xu J. Y., Liu D., Zhong Y., Huang R. C. (2017). Effects of timing on intracoronary autologous bone marrow-derived cell transplantation in acute myocardial infarction: a meta-analysis of randomized controlled trials. *Stem Cell Research & Therapy*.

[23] Li R., Li X.-M., Chen J.-R. (2016). Clinical efficacy and safety of autologous stem cell trans-plantation for patients with ST-segment elevation myocardial infarction. *Therapeutics and Clinical Risk Management*.

[24] Jeong H., Yim H. W., Park H.-J. (2018). Mesenchymal stem cell therapy for ischemic heart disease: systematic review and meta-analysis. *International Journal of Stem Cells*.

[25] Hare J. M., Traverse J. H., Henry T. D. (2009). A randomized, double-Blind, placebo-controlled, dose-escalation study of intravenous adult human mesenchymal stem cells (prochymal) after acute myocardial infarction. *Journal of the American College of Cardiology*.

[26] Penn M. S., Ellis S., Gandhi S. (2012). Adventitial delivery of an allogeneic bone marrow derived adherent stem cell in acute myocardial infarction: phase I clinical study. *Circulation Research*.

[27] Gao L. R., Pei X. T., Ding Q. A. (2013). A critical challenge: dosage-related efficacy and acute complication intracoronary injection of autologous bone marrow mesenchymal stem cells in acute myocardial infarction. *International Journal of Cardiology*.

[28] Traverse J. H., Henry T. D., Pepine C. J., Willerson J. T., Ellis S. G. (2014). One-year follow-up of intracoronary stem cell delivery on left ventricular function following ST-elevation myocardial infarction. *JAMA*.

[29] Hu X., Huang X., Yang Q. (2015). Safety and efficacy of intracoronary hypoxia preconditioned bone marrow mononuclear cell administration for acute myocardial infarction patients: the CHINA-AMI randomized controlled trial. *International Journal of Cardiology*.

[30] Hare J. M., Fishman J. E., Gerstenblith G. (2012). Comparison of allogeneic vs autologous bone marrow–derived mesenchymal stem cells delivered by transendocardial injection in patients with ischemic cardiomyopathy: the POSEIDON randomized trial. *JAMA*.

[31] Suncion V. Y., Ghersin E., Fishman J. E. (2014). Does transendocardial injection of mesenchymal stem cells improve myocardial function locally or globally?. *Circulation Research*.

[32] Can A., Ulus A. T., Cinar O. (2015). Human umbilical cord mesenchymal stromal cell transplantation in myocardial ischemia (HUC-HEART Trial). A study protocol of a phase 1/2, controlled and randomized trial in combination with coronary artery bypass grafting. *Stem Cell Reviews and Reports*.

[33] Chullikana A., Majumdar A. S., Gottipamula S. (2015). Randomized, double-blind , phase I/II study of intravenous allogeneic mesenchymal stromal cells in acute myocardial infarction. *Cytotherapy*.

[34] Schutt R. C., Trachtenberg B. H., Cooke J. P. (2015). Bone marrow characteristics associated wi-th changes in infarct size after STEMI: a biorepository evaluation from the CCTRN TIME trial. *Circulation Research*.

[35] San Roman J. A., Sánchez P. L., Villa A. (2015). Comparison of different bone marrow–derived stem cell approaches in reperfused STEMI a multicenter, prospective, randomized, open-labeled TECAM trial. *Journal of the American College of Cardiology*.

[36] Choudry F., Hamshere S., Saunders N. (2016). A randomized double-blind control study of early intra-coronary autologous bone marrow cell infusion in acute myocardial infarction : the REGENERATE-AMI clinical trial. *European Heart Journal*.

[37] Gao L. R., Chen Y., Zhang N. K. (2015). Intracoronary infusion of Wharton’s jelly-derived mesenchymal stem cells in acute myocardial infarction: double-blind, randomized controlled trial. *BMC Medicine*.

[38] Florea V., Rieger A. C., DiFede D. L. (2017). Dose comparison study of allogeneic mesenchymal stem cells in patients with ischemic cardiomyopathy (The TRIDENT study). *Circulation Research*.

[39] Lee J. W., Lee S. H., Youn Y. J. (2014). A randomized, open-label, multicenter trial for the safety and efficacy of adult mesenchymal stem cells after acute myocardial infarction. *Journal of Korean Medical Science*.

[40] Elahi K. C., Klein G., Avci-Adali M., Sievert K. D., MacNeil S., Aicher W. K. (2016). Human mesenchymal stromal cells from different sources diverge in their expression of cell surface proteins and display distinct differentiation patterns. *Stem Cells International*.

[41] Liang H., Hou H., Yi W. (2013). Increased expression of pigment epithelium-derived factor in aged mesenchymal stem cells impairs their therapeutic efficacy for attenuating myocardial infarction injury. *European Heart Journal*.

[42] Haque N., Rahman M. T., Abu Kasim N. H., Alabsi A. M. (2013). Hypoxic culture conditions as a solution for mesenchymal stem cell based regenerative therapy. *The Scientific World Journal*.

[43] Ren G., Chen X., Dong F. (2012). Concise review: mesenchymal stem cells and translational medicine: emerging issues. *Stem Cells Translational Medicine*.

[44] Marquez-Curtis L. A., Janowska-Wieczorek A. (2013). Enhancing the migration ability of mesenchymal stromal cells by targeting the SDF-1/CXCR4 axis. *BioMed Research International*.

[45] Karpov A. A., Udalova D. V., Pliss M. G., Galagudza M. M. (2017). Can the outcomes of mesenchymal stem cell-based therapy for myocardial infarction be improved? Providing weapons and armour to cells. *Cell Proliferation*.

[46] Haque N., Kasim N. H. A., Rahman M. T. (2015). Optimization of pre-transplantation conditions to enhance the efficacy of mesenchymal stem cells. *International Journal of Biological Sciences*.

[47] Hu X., Xu Y., Zhong Z. (2016). A large-scale investigation of hypoxia-preconditioned allogeneic mesenchymal stem cells for myocardial repair in nonhuman primates: paracrine activity without remuscularization. *Circulation Research*.

[48] Liu Y., Yang X., Maureira P. (2017). Permanently hypoxic cell culture yields rat bone marrow mesenchymal cells with higher therapeutic potential in the treatment of chronic myocardial infarction. *Cellular Physiology and Biochemistry*.

[49] Saini U., Gumina R. J., Wolfe B., Kuppusamy M. L., Kuppusamy P., Boudoulas K. D. (2013). Preconditioning mesenchymal stem cells with caspase inhibition and hyperoxia prior to hypoxia exposure increases cell proliferation. *Journal of Cellular Biochemistry*.

[50] Zhang J., Chen G. H., Wang Y. W. (2012). Hydrogen peroxide preconditioning enhances the therapeutic efficacy of Wharton’s Jelly mesenchymal stem cells after myocardial infarction. *Chinese Medical Journal*.

[51] Qiao P. F., Yao L., Zhang X. C., Li G. D., Wu D. Q. (2015). Heat shock pretreatment improves stem cell repair following ischemia-reperfusion injury via autophagy. *World Journal of Gastroenterology*.

[52] Moya A., Larochette N., Paquet J. (2017). Quiescence preconditioned human multipotent stromal cells adopt a metabolic profile favorable for enhanced survival under ischemia. *Stem Cells*.

[53] Baldari S., di Rocco G., Piccoli M., Pozzobon M., Muraca M., Toietta G. (2017). Challenges and strategies for improving the regenerative effects of mesenchymal stromal cell-based therapies. *International Journal of Molecular Sciences*.

[54] Flynn A., Chen X., O'connell E., O'brien T. (2012). A comparison of the efficacy of transplantation of bone marrow-derived mesenchymal stem cells and unrestricted somatic stem cells on outcome after acute myocardial infarction. *Stem Cell Research & Therapy*.

[55] Han S. M., Han S. H., Coh Y. R. (2014). Enhanced proliferation and differentiation of Oct 4- and Sox2-overexpressing human adipose tissue mesenchymal stem cells. *Experimental & Molecular Medicine*.

[56] Baldari S., di Rocco G., Trivisonno A., Samengo D., Pani G., Toietta G. (2016). Promotion of survival and engraftment of transplanted adipose tissue-derived stromal and vascular cells by overexpression of manganese superoxide dismutase. *International Journal of Molecular Sciences*.

[57] Caroti C. M., Ahn H., Salazar H. F. (2017). A novel technique for accelerated culture of murine mesenchymal stem cells that allows for sustained multipotency. *Scientific Reports*.

[58] Liu X.-B., Wang J.-A., Ji X.-Y., Yu S., Wei L. (2014). Preconditioning of bone marrow mesenchymal stem cells by prolyl hydroxylase inhibition enhances cell survival and angiogenesis in vitro and after transplantation into the ischemic heart of rats. *Stem Cell Research & Therapy*.

[59] Wei Z. Z., Zhu Y. B., Zhang J. Y. (2017). Priming of the cells: hypoxic preconditioning for stem cell therapy. *Chinese Medical Journal*.

[60] McGinley L. M., McMahon J., Stocca A. (2013). Mesenchymal stem cell survival in the infarcted heart is enhanced by lentivirus vector-mediated heat shock protein 27 expression. *Human Gene Therapy*.

[61] Chen Y., Zhao Y., Chen W. (2017). MicroRNA-133 overexpression promotes the therapeutic efficacy of mesenchymal stem cells on acute myocardial infarction. *Stem Cell Research & Therapy*.

[62] Su G., Liu L., Yang L., Mu Y., Guan L. (2018). Homing of endogenous bone marrow mesenchymal stem cells to rat infarcted myocardium via ultrasound-mediated recombinant SDF-1*α* adenovirus in microbubbles. *Oncotarget*.

[63] Dong F., Patnaik S., Duan Z. H., Kiedrowski M., Penn M. S., Mayorga M. E. (2017). A novel role for CAMKK1 in the regulation of the mesenchymal stem cell secretome. *Stem Cells Translational Medicine*.

[64] Chen L., Zhang Y., Tao L., Yang Z., Wang L. (2017). Mesenchymal stem cells with eNOS over-expression enhance cardiac repair in rats with myocardial infarction. *Cardiovascular Drugs and Therapy*.

[65] Wang L., Pasha Z., Wang S. (2013). Protein kinase G1 *α* overexpression increases stem cell survival and cardiac function after myocardial infarction. *PLoS One*.

[66] Liang Y., Lin Q., Zhu J. (2014). The caspase-8 shRNA-modified mesenchymal stem cells improve the function of infarcted heart. *Molecular and Cellular Biochemistry*.

[67] Liu X., Chen H., Zhu W. (2014). Transplantation of SIRT1-engineered aged mesenchymal stem cells improves cardiac function in a rat myocardial infarction model. *The Journal of Heart and Lung Transplantation*.

[68] Ke T., Wu Y., Li L. (2014). Netrin-1 ameliorates myocardial infarction-induced myocardial injury: mechanisms of action in rats and diabetic mice. *Human Gene Therapy*.

[69] Chen X. Q., Chen L. L., Fan L., Fang J., Chen Z. Y., Li W. W. (2014). Stem cells with FGF4-bFGF fused gene enhances the expression of bFGF and improves myocardial repair in rats. *Biochemical and Biophysical Research Communications*.

[70] Wen Z., Huang W., Feng Y. (2014). MicroRNA-377 regulates mesenchymal stem cell-induced angiogenesis in ischemic hearts by targeting VEGF. *PLoS One*.

[71] He H., Zhao Z. H., Han F. S., Liu X. H., Wang R., Zeng Y. J. (2016). Overexpression of protein kinase C *ɛ* improves retention and survival of transplanted mesenchymal stem cells in rat acute myocardial infarction. *Cell Death and Disease*.

[72] Yang C. J., Yang J., Yang J., Fan Z. X. (2016). Thioredoxin-1 (Trx1) engineered mesenchymal stem cell therapy is a promising feasible therapeutic approach for myocardial infarction. *International Journal of Cardiology*.

[73] Xue X., Liu Y., Zhang J., Liu T., Yang Z., Wang H. (2015). Bcl-xL genetic modification enhanced the therapeutic efficacy of mesenchymal stem cell transplantation in the treatment of heart infarction. *Stem Cells International*.

[74] Zhao S. L., Zhang Y. J., Li M. H., Zhang X. L., Chen S. L. (2014). Mesenchymal stem cells with overexpression of midkine enhance cell survival and attenuate cardiac dysfunction in a rat model of myocardial infarction. *Stem Cell Research & Therapy*.

[75] Mao J., Lv Z., Zhuang Y. (2014). MicroRNA-23a is involved in tumor necrosis factor-*α* induced apoptosis in mesenchymal stem cells and myocardial infarction. *Experimental and Molecular Pathology*.

[76] Ham O., Lee S. Y., Lee C. Y. (2015). Let-7b suppresses apoptosis and autophagy of human mesenchymal stem cells transplanted into ischemia/reperfusion injured heart 7by targeting caspase-3. *Stem Cell Research & Therapy*.

[77] Moon H. H., Joo M. K., Mok H. (2014). MSC-based VEGF gene therapy in rat myocardial infarction model using facial amphipathic bile acid-conjugated polyethyleneimine. *Biomaterials*.

[78] Cerrada I., Ruiz-Saurí A., Carrero R. (2013). Hypoxia-inducible factor 1 alpha contributes to cardiac healing in mesenchymal stem cells-mediated cardiac repair. *Stem Cells and Development*.

[79] Gao L., Bledsoe G., Yin H., Shen B., Chao L., Chao J. (2013). Tissue kallikrein-modified mesenchymal stem cells provide enhanced protection against ischemic cardiac injury after myocardial infarction. *Circulation Journal*.

[80] Zhu K., Lai H., Guo C. (2014). Nanovector-based prolyl hydroxylase domain 2 silencing system enhances the efficiency of stem cell transplantation for infarcted myocardium repair. *International Journal of Nanomedicine*.

[81] Pan Q., Qin X., Ma S. (2014). Myocardial protective effect of extracellular superoxide dismutase gene modified bone marrow mesenchymal stromal cells on infarcted mice hearts. *Theranostics*.

[82] Huang F., Li M. L., Fang Z. F. (2013). Overexpression of MicroRNA-1 improves the efficacy of mesenchymal stem cell transplantation after myocardial infarction. *Cardiology*.

[83] Zhao L., Liu X., Zhang Y. (2016). Enhanced cell survival and paracrine effects of mesenchymal stem cells overexpressing hepatocyte growth factor promote cardioprotection in myocardial infarction. *Experimental Cell Research*.

[84] Mao Q., Lin C., Gao J. (2014). Mesenchymal stem cells overexpressing integrin-linked kinase attenuate left ventricular remodeling and improve cardiac function after myocardial infarction. *Molecular and Cellular Biochemistry*.

[85] Gómez-Mauricio G., Moscoso I., Martín-Cancho M.-F. (2016). Combined administration of mesenchymal stem cells overexpressing IGF-1 and HGF enhances neovascularization but moderately improves cardiac regeneration in a porcine model. *Stem Cell Research & Therapy*.

[86] de Jong R., van Hout G. P. J., Houtgraaf J. H. (2014). Intracoronary infusion of encapsulated glucagon-like peptide-1-eluting mesenchymal stem cells preserves left ventricular function in a porcine model of acute myocardial infarction. *Circulation. Cardiovascular Interventions*.

[87] Locatelli P., Olea F. D., Hnatiuk A. (2015). Mesenchymal stromal cells overexpressing vascular endothelial growth factor in ovine myocardial infarction. *Gene Therapy*.

[88] Xu J., Huang Z., Lin L. (2014). miR-210 over-expression enhances mesenchymal stem cell survival in an oxidative stress environment through antioxidation and c-Met pathway activation. *Science China Life Sciences*.

[89] Xiaowei C., Jia M., Xiaowei W., Yina Z. (2013). Overexpression of CXCL12 chemokine up-regulates connexin and integrin expression in mesenchymal stem cells through PI3K/Akt pathway. *Cell Communication & Adhesion*.

[90] Hou J., Wang L., Hou J. (2015). Peroxisome proliferator-activated receptor gamma promotes mesenchymal stem cells to express connexin43 via the inhibition of TGF-*β*1/Smads signaling in a rat model of myocardial infarction. *Stem Cell Reviews*.

[91] Li N., Yang Y. J., Qian H. Y. (2015). Intravenous administration of atorvastatin-pretreated mesenchymal stem cells improves cardiac performance after acute myocardial infarction role of CXCR4. *American Journal of Translational Research*.

[92] Elmadbouh I., Ashraf M. (2017). Tadalafil, a long acting phosphodiesterase inhibitor, promotes bone marrow stem cell survival and their homing into ischemic myocardium for cardiac repair. *Physiological Reports*.

[93] Gong X. (2016). Protective effect of *ailanthus excelsa* roxb in myocardial infarction post mesenchymal stem cell transplantation: study in chronic ischemic rat model. *African Journal of Traditional, Complementary, and Alternative Medicines*.

[94] Liu X., Hu D., Zeng Z. (2017). SRT1720 promotes survival of aged human mesenchymal stem cells via FAIM: a pharmacological strategy to improve stem cell-based therapy for rat myocardial infarction. *Cell Death & Disease*.

[95] Liu C., Fan Y., zhou L. (2015). Pretreatment of mesenchymal stem cells with angiotensin II enhances paracrine effects, angiogenesis, gap junction formation and therapeutic efficacy for myocardial infarction. *International Journal of Cardiology*.

[96] Guo H. D., Cui G. H., Tian J. X. (2014). Transplantation of salvianolic acid B pretreated mesenchymal stem cells improves cardiac function in rats with myocardial infarction through angiogenesis and paracrine mechanisms. *International Journal of Cardiology*.

[97] Khan I., Ali A., Akhter M. A. (2016). Preconditioning of mesenchymal stem cells with 2,4-dinitrophenol improves cardiac function in infarcted rats. *Life Sciences*.

[98] Zhang G. W., Gu T. X., Sun X. J. (2016). Edaravone promotes activation of resident cardiac stem cells by transplanted mesenchymal stem cells in a rat myocardial infarction model. *The Journal of Thoracic and Cardiovascular Surgery*.

[99] Xu H., Zhu G., Tian Y. (2012). Protective effects of trimetazidine on bone marrow mesenchymal stem cells viability in an *ex vivo* model of hypoxia and *in vivo* model of locally myocardial ischemia. *Journal of Huazhong University of Science and Technology. Medical Sciences*.

[100] Guo J., Zheng D., Li W. F., Li H. R., Zhang A. D., Li Z. C. (2014). Insulin-like growth factor 1 treatment of MSCs attenuates inflammation and cardiac dysfunction following MI. *Inflammation*.

[101] Wang C.-M., Guo Z., Xie Y. J. (2014). Co-treating mesenchymal stem cells with IL-1*β* and TNF-*α* increases VCAM-1 expression and improves post-ischemic myocardial function. *Molecular Medicine Reports*.

[102] Liu Y.-L., Zhou Y., Sun L. (2014). Protective effects of gingko biloba extract 761 on myocardial infarction via improving the viability of implanted mesenchymal stem cells in the rat heart. *Molecular Medicine Reports*.

[103] Ye L., Zhang P., Duval S., Su L., Xiong Q., Zhang J. (2013). Thymosin *β*4 increases the potency of transplanted mesenchymal stem cells for myocardial repair. *Circulation*.

[104] Xie J., Wang H., Song T. (2013). Tanshinone IIA and astragaloside IV promote the migration of mesenchymal stem cells by up-regulation of CXCR4. *Protoplasma*.

[105] Han D., Huang W., Li X. (2016). Melatonin facilitates adipose-derived mesenchymal stem cells to repair the murine infarcted heart via the SIRT1 signaling pathway. *Journal of Pineal Research*.

[106] Cho D. I., Kang W. S., Hong M. H. (2017). The optimization of cell therapy by combinational application with apicidin-treated mesenchymal stem cells after myocardial infarction. *Oncotarget*.

[107] Lu W., Xie Z., Tang Y. (2015). Photoluminescent mesoporous silicon nanoparticles with siCCR2 improve the effects of mesenchymal stromal cell transplantation after acute myocardial infarction. *Theranostics*.

[108] Franchi F., Ezenekwe A., Wellkamp L., Peterson K. M., Lerman A., Rodriguez-Porcel M. (2014). Renin inhibition improves the survival of mesenchymal stromal cells in a mouse model of myocardial infarction. *Journal of Cardiovascular Translational Research*.

[109] Song L., Yang Y. J., Dong Q. T. (2013). Atorvastatin enhance efficacy of mesenchymal stem cells treatment for swine myocardial infarction via activation of nitric oxide synthase. *PLoS One*.

[110] Hsu W. T., Jui H. Y., Huang Y. H. (2015). CXCR4 antagonist TG-0054 mobilizes mesenchymal stem cells, attenuates inflammation, and preserves cardiac systolic function in a porcine model of myocardial infarction. *Cell Transplantation*.

[111] Wright E. J., Hodson N. W., Sherratt M. J. (2016). Combined MSC and GLP-1 therapy modulates collagen remodeling and apoptosis following myocardial infarction. *Stem Cells International*.

[112] Yang J., Xia J., He Y., Zhao J., Zhang G. (2015). MSCs transplantation with application of G-CSF reduces apoptosis or increases VEGF in rabbit model of myocardial infarction. *Cytotechnology*.

[113] Qu Z., Xu H., Tian Y., Jiang X. (2013). Atorvastatin improves microenvironment to enhance the beneficial effects of BMSCs therapy in a rabbit model of acute myocardial infarction. *Cellular Physiology and Biochemistry*.

[114] Zhang F., Cui J., Lv B., Yu B. (2015). Nicorandil protects mesenchymal stem cells against hypoxia and serum deprivation-induced apoptosis. *International Journal of Molecular Medicine*.

[115] Huang D., Yin L., Liu X. (2017). Geraniin protects bone marrow-derived mesenchymal stem cells against hydrogen peroxide-induced cellular oxidative stress in vitro. *International Journal of Molecular Medicine*.

[116] Zhou H., Li D., Shi C. (2015). Effects of exendin-4 on bone marrow mesenchymal stem cell proliferation, migration and apoptosis in vitro. *Scientific Reports*.

[117] Pourrajab F., Babaei Zarch M., Baghi Yazdi M., Rahimi Zarchi A., Vakili Zarch A. (2014). Application of stem cell/growth factor system, as a multimodal therapy approach in regenerative medicine to improve cell therapy yields. *International Journal of Cardiology*.

[118] Yukawa H., Watanabe M., Kaji N. (2012). Monitoring transplanted adipose tissue-derived stem cells combined with heparin in the liver by fluorescence imaging using quantum dots. *Biomaterials*.

[119] Dollinger B. R., Gupta M. K., Martin J. R., Duvall C. L. (2017). Reactive oxygen species shielding hydrogel for the delivery of adherent and nonadherent therapeutic cell types. *Tissue Engineering Part A*.

[120] Sürder D., Manka R., Moccetti T. (2016). Effect of bone marrow-derived mononuclear cell treatment, early or late after acute myocardial infarction: twelve months CMR and long-term clinical results. *Circulation Research*.

[121] Lamirault G., Susen S., Forest V. (2013). Difference in mobilization of progenitor cells after myocardial infarction in smoking versus non-smoking patients: insights from the BONAMI trial. *Stem Cell Research & Therapy*.

[122] Zhang Z., Li S., Cui M. (2013). Rosuvastatin enhances the therapeutic efficacy of adipose-derived mesenchymal stem cells for myocardial infarction via PI3K/Akt and MEK/ERK pathways. *Basic Research in Cardiology*.

[123] Tobita M., Tajima S., Mizuno H. (2015). Adipose tissue-derived mesenchymal stem cells and platelet-rich plasma: stem cell transplantation methods that enhance stemness. *Stem Cell Research & Therapy*.

[124] Tsilimigras D. I., Oikonomou E. K., Moris D., Schizas D., Economopoulos K. P., Mylonas K. S. (2017). Stem cell therapy for congenital heart disease: a systematic review. *Circulation*.

[125] Ichihara Y., Kaneko M., Yamahara K. (2018). Self-assembling peptide hydrogel enables instant epicardial coating of the heart with mesenchymal stromal cells for the treatment of heart failure. *Biomaterials*.

[126] Golpanian S., Schulman I. H., Ebert R. F. (2016). Concise review: review and perspective of cell dosage and routes of administration from preclinical and clinical studies of stem cell therapy for heart disease. *Stem Cells Translational Medicine*.

[127] Ottersbach A., Mykhaylyk O., Heidsieck A. (2018). Improved heart repair upon myocardial infarction: combination of magnetic nanoparticles and tailored magnets strongly increases engraftment of myocytes. *Biomaterials*.

[128] Cesarz Z., Tamama K. (2016). Spheroid culture of mesenchymal stem cells. *Stem Cells International*.

[129] Murphy K. C., Fang S. Y., Leach J. K. (2014). Human mesenchymal stem cell spheroids in fibrin hydrogels exhibit improved cell survival and potential for bone healing. *Cell and Tissue Research*.

[130] Cheng N. C., Wang S., Young T. H. (2012). The influence of spheroid formation of human adipose-derived stem cells on chitosan films on stemness and differentiation capabilities. *Biomaterials*.

[131] Bhang S. H., Lee S., Shin J. Y., Lee T. J., Kim B. S. (2012). Transplantation of cord blood mesenchymal stem cells as spheroids enhances vascularization. *Tissue Engineering. Part A*.

[132] Lee E. J., Park S. J., Kang S. K. (2012). Spherical bullet formation via E-cadherin promotes therapeutic potency of mesenchymal stem cells derived from human umbilical cord blood for myocardial infarction. *Molecular Therapy*.

[133] Emmert M. Y., Wolint P., Wickboldt N. (2013). Human stem cell-based three-dimensional microtissues for advanced cardiac cell therapies. *Biomaterials*.

[134] Tanaka Y., Shirasawa B., Takeuchi Y. (2016). Autologous preconditioned mesenchymal stem cell sheets improve left ventricular function in a rabbit old myocardial infarction model. *American Journal of Translational Research*.

[135] Assmus B., Leistner D. M., Schächinger V. (2014). Long-term clinical outcome after intracoronary application of bone marrow-derived mononuclear cells for acute myocardial infarction: migratory capacity of administered cells determines event-free survival. *European Heart Journal*.

[136] Aguado B. A., Mulyasasmita W., Su J., Lampe K. J., Heilshorn S. C. (2012). Improving viability of stem cells during syringe needle flow through the design of hydrogel cell carriers. *Tissue Engineering. Part A*.

[137] Bernhard J. C., Vunjak-Novakovic G. (2016). Should we use cells, biomaterials, or tissue engineering for cartilage regeneration?. *Stem Cell Research & Therapy*.

[138] Follin B., Juhl M., Cohen S. (2015). Human adipose-derived stromal cells in a clinically applicable injectable alginate hydrogel: phenotypic and immunomodulatory evaluation. *Cytotherapy*.

[139] Levit R. D., Landázuri N., Phelps E. A. (2013). Cellular encapsulation enhances cardiac repair. *Journal of the American Heart Association*.

[140] Martino M. M., Briquez P. S., Guc E. (2014). Growth factors engineered for super-affinity to the extracellular matrix enhance tissue healing. *Science*.

[141] Rodrigo S. F., van Ramshorst J., Hoogslag G. E. (2013). Intramyocardial injection of autologous bone marrow-derived ex vivo expanded mesenchymal stem cells in acute myocardial infarction patients is feasible and safe up to 5 years of follow-up. *Journal of Cardiovascular Translational Research*.

[142] Li N., Huang R., Zhang X. (2017). Stem cells cardiac patch from decellularized umbilical artery improved heart function after myocardium infarction. *Bio-medical Materials and Engineering*.

[143] Ravichandran R., Venugopal J. R., Mukherjee S., Sundarrajan S., Ramakrishna S. (2015). Elastomeric core/shell nanofibrous cardiac patch as a biomimetic support for infarcted porcine myocardium. *Tissue Engineering. Part A*.

[144] Li X., Chen Y. Y., Wang X. M. (2017). Image-guided stem cells with functionalized self-assembling peptide nanofibers for treatment of acute myocardial infarction in a mouse model. *American Journal of Translational Research*.

[145] Han J., Park J., Kim B. S. (2015). Integration of mesenchymal stem cells with nanobiomaterials for the repair of myocardial infarction. *Advanced Drug Delivery Reviews*.

[146] Ma Q., Yang J., Huang X. (2018). Poly(lactide-co-glycolide)-monomethoxy-poly-(polyethylene glycol) nanoparticles loaded with melatonin protect adipose-derived stem cells transplanted in infarcted heart tissue. *Stem Cells*.

[147] Yan W., Guo Y., Tao L. (2017). C1q/tumor necrosis factor–related protein-9 regulates the fate of implanted mesenchymal stem cells and mobilizes their protective effects against ischemic heart injury via multiple novel signaling pathways. *Circulation*.

[148] Becker A. D., Riet I. V. (2016). Homing and migration of mesenchymal stromal cells: how to improve the efficacy of cell therapy?. *World Journal of Stem Cells*.

[149] Kean T. J., Duesler L., Young R. G. (2011). Development of a peptide-targeted, myocardial ischemia-homing, mesenchymal stem cell. *Journal of Drug Targeting*.

[150] El Gammal Z. H., Zaher A. M., El-Badri N. (2017). Effect of low-level laser-treated mesenchymal stem cells on myocardial infarction. *Lasers in Medical Science*.

[151] Li L., Wu S., Liu Z. (2015). Ultrasound-targeted microbubble destruction improves the migration and homing of mesenchymal stem cells after myocardial infarction by upregulating SDF-1/CXCR4: a pilot study. *Stem Cells International*.

[152] Huang Z., Shen Y., Sun A. (2013). Magnetic targeting enhances retrograde cell retention in a rat model of myocardial infarction. *Stem Cell Research & Therapy*.

